# Comparison of Hysterosalpingography With Laparoscopy in the Diagnosis of Tubal Factor of Female Infertility

**DOI:** 10.3389/fmed.2021.720401

**Published:** 2021-10-29

**Authors:** Jifan Tan, Min Deng, Meng Xia, Muhua Lai, Wenwei Pan, Yubin Li

**Affiliations:** ^1^Reproductive Medicine Center, The First Affiliated Hospital, Sun Yat-sen University, Guangzhou, China; ^2^Guangdong Provincial Key Laboratory of Reproductive Medicine, Guangzhou, China; ^3^Department of Obstetrics and Gynecology, The First Affiliated Hospital, Sun Yat-sen University, Guangzhou, China

**Keywords:** hysterosalpingography, laparoscopy, infertility, diagnose, fallopian tubes

## Abstract

**Background:** Laparoscopy is considered to be the gold standard in the evaluation of causes leading to infertility. Hysterosalpingography (HSG) permits indirect visualization of the cervical canal, uterine cavity, and tube patency, which is helpful for evaluating the causes of infertility.

**Objective:** This study aimed to detect tubal abnormalities in infertile women by HSG or laparoscopy and determine the value of HSG in diagnosing fallopian tube status.

**Methods:** The study group consisted of 1,276 patients. HSG was performed as a preliminary test for the evaluation of fallopian tube status. Women were subjected to laparoscopic examination on evidence of HSG abnormalities.

**Results:** The negative predictive value of HSG for detecting patency or occlusion for the right/left tube was 92.08 and 95.44%, respectively. The kappa values for the consistent diagnosis in the right/left tube were 0.470 and 0.574, respectively. In cases of low patency of the right/left tube, there was a greater than a 40% chance for the tube to be patent, and the remaining high probability was pelvic adhesion. The positive predictive value of HSG for detecting patency or occlusion for both tubes was 87.2%. The kappa value was 0.898 [95% CI (0.838, 0.937), *p* < 0.001], which meant that the diagnostic accuracy of HSG for both tube patency/occlusion was explicit. The kappa value for the diagnosis of hydrosalpinx (especially for bilateral tube hydrosalpinx) was 0.838 [95% CI (0.754, 0.922), *p* < 0.001], and the diagnostic accuracy for HSG was 79.8, 67.9, and 72.4%, respectively.

**Conclusion:** The current study concluded that HSG is a good diagnostic modality to detect tube abnormalities in infertile patients. HSG and laparoscopy are complementary to each other and whenever the patient is undertaken for diagnosis of infertility. Cost-effective HSG had good predictive value in identifying tubal factor infertility.

## Introduction

Infertility is defined as a failure of conception in a couple who has a regular unprotected sexual activity for 1 year and still does not conceive (1). Many factors can result in infertility, including disorder in fallopian tubes, anovulation, and pelvic adhesion leading to pelvic microenvironments. Among the factors mentioned above, disorders of the fallopian tube account for 30–45% of the reasons for infertility (2, 3). Hence, screening for tubal occlusion is one of the first essential steps of infertility assessment. In recent years, with the development of endoscopic techniques, the diagnosis and treatment of female infertility have made significant advances (3, 4).

Hysterosalpingography (HSG) is a contrast-enhanced fluoroscopic radiological technique adopted to evaluate the uterine cavity, fallopian tubes, and adjacent peritoneum after injection of contrast media through the cervical canal (5). It determines the patency of fallopian tubes, the contour of the uterus, and the adjacent pelvic peritoneum in patients experiencing assessment for infertility. Sometimes, HSG gives us the first indication for the underlying reasons leading to infertility (6, 7).

Although HSG provides us with a permanent record of the fluoroscopic examination of the uterine cavity and tubal patency, subtle changes such as pelvic adhesions and endometriosis, which influence fertility without any pelvic anatomy changes, can be missed (7–9). Laparoscopy can magnify some subtle differences in the fallopian tube or pelvic peritoneum. Although it was considered the “gold standard” procedure for determining the reasons for infertility (10–12), it was not recommended as the first-line clinical evaluation test because it is an invasive procedure also needing anesthesia, thus adding to the cost and side effects.

This study aimed to compare the diagnostic value of HSG in evaluating tubal patency and pelvic adhesion in the hope of providing some clinical value in the diagnosis of infertility.

## Materials and Methods

### Patients

From January 2014 to November 2020, we retrospectively studied 1,276 patients who underwent HSG or laparoscopic examination for infertility. First, HSG was performed. If the results of HSG were normal or not patent, but the patients did not become pregnant in the 12 months after examination, we performed a laparoscopic procedure. If the results of HSG were occlusion or hydrosalpinx, but the patients desired to conceive, naturally, they chose to perform the laparoscopic examination. All the enrolled patients had a regular menstrual cycle, and routine semen examination of the husband was normal. We excluded patients who had an ovarian cyst, uterine malformation, endometriosis, or any other type of organic lesion that could be found by routine ultrasonography. The medical ethics committee of the First Affiliated Hospital of Sun Yat-sen University approved the study.

### HSG Examination

**Figure 1:**
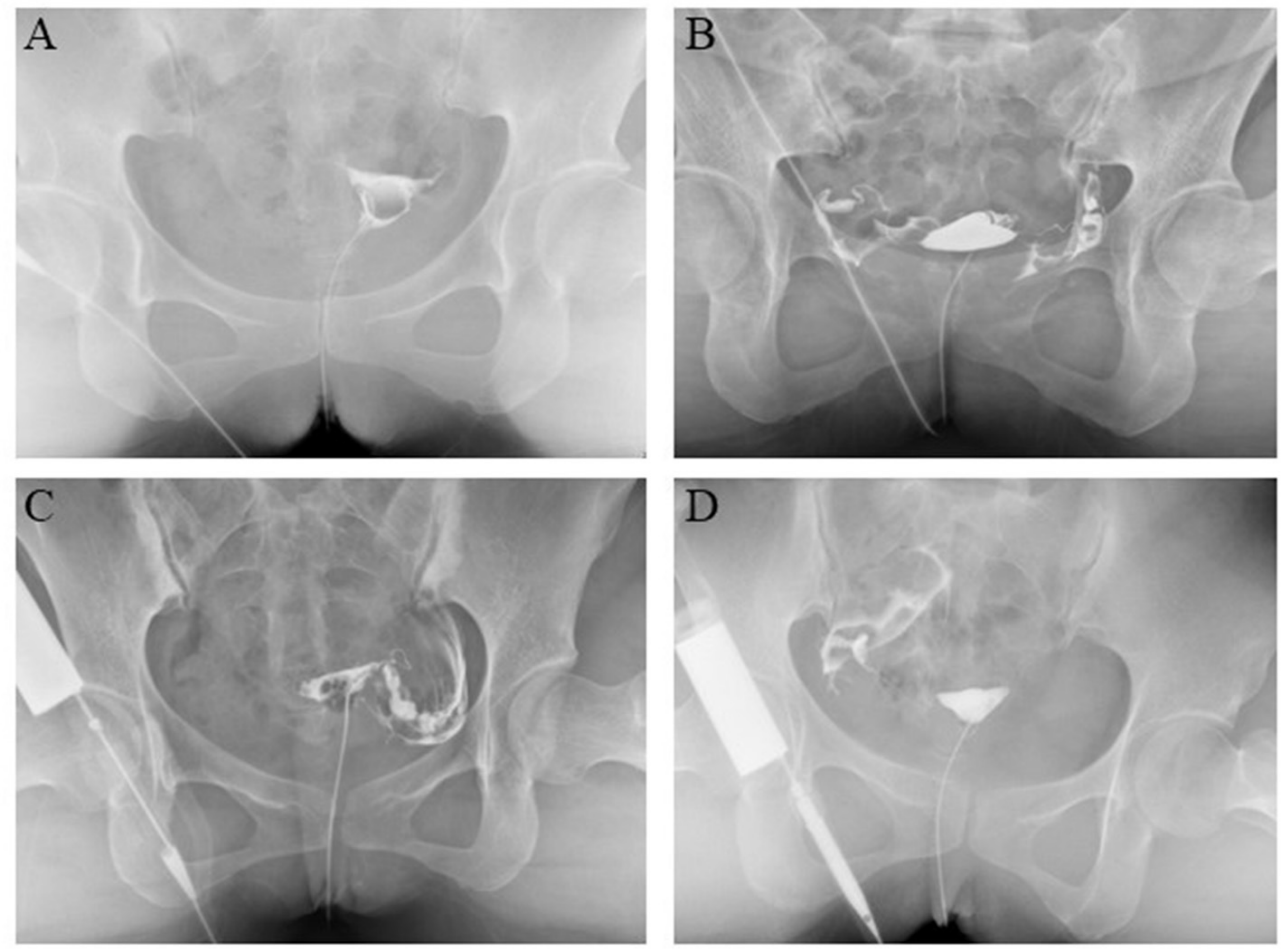
Diagnosis of fallopian tube patency or occlusion by hysterosalpingography (HSG). **(A)** Both fallopian tube patency. **(B)** Both fallopian tube occlusion. **(C)** Left tube patency and right tube occlusion. **(D)** Right tube patency and left tube occlusion.

**Figure 2:**
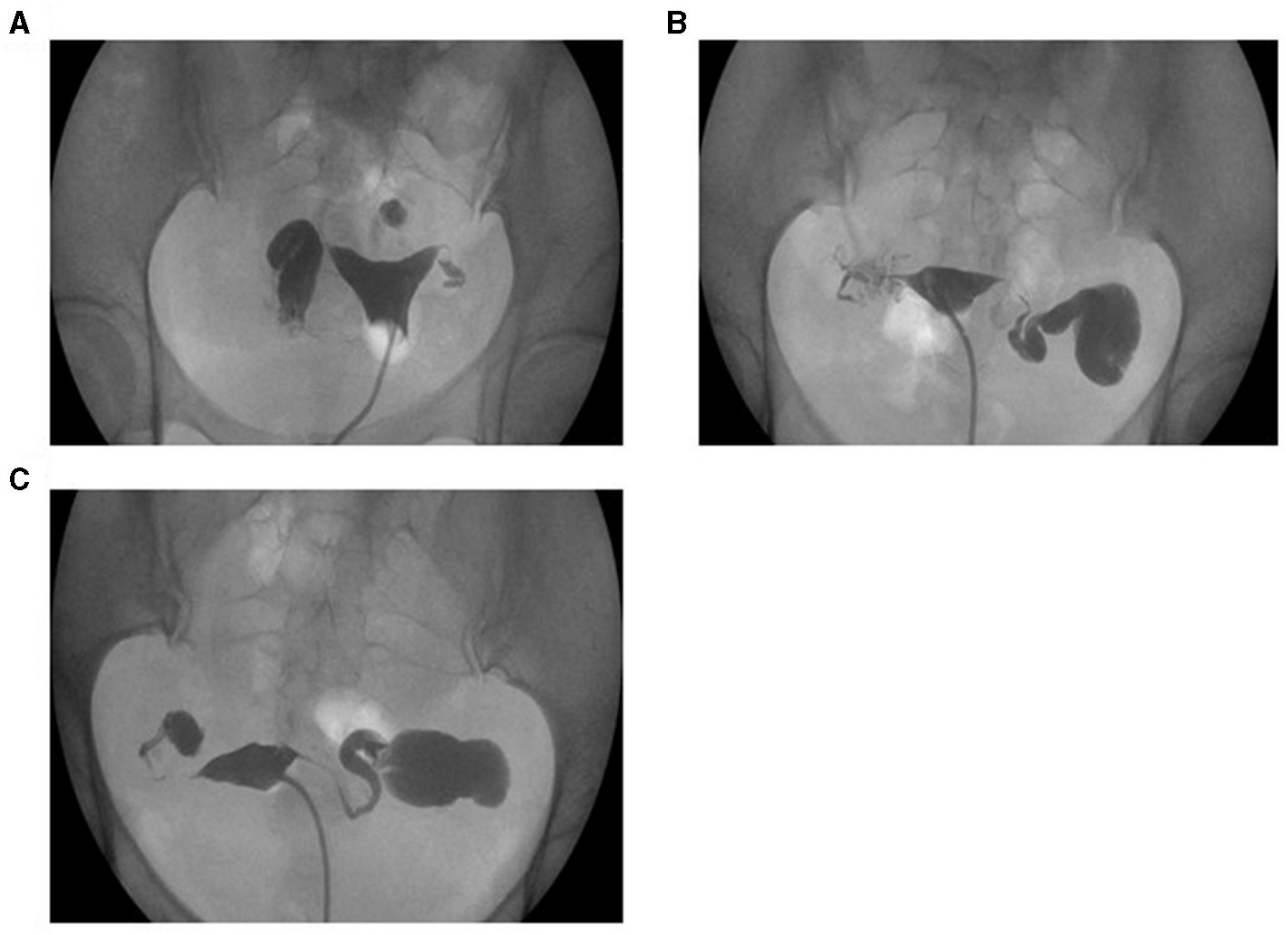
Diagnosis of hydrosalpinx by HSG. **(A)** Right tube hydrosalpinx. **(B)** Left tube hydrosalpinx. **(C)** Both tube hydrosalpinx.

Hysterosalpingography examination was performed 3–7 days after menstruation. An experienced technician performed the procedures, and two separate radiologists determined the results. According to the image, the patency of the tube could be divided into no patency, patency, and occlusion (Figure 1). If combined with hydrosalpinx (Figure 2), the diagnosis would then be added. The criteria for low patency were the following: the iodine agent in the whole oviduct was absorbent, but the lumen wall was rough, thickened, narrow, and knotted, or the iodine agent remained for 24 h.

### Laparoscopic Examination

The laparoscopic examination was performed 7–12 days after menstruation. The patients underwent this procedure with general anesthesia. During the process, we directly found pelvic adhesion, and methylene blue staining was used to determine the patency of the fallopian tube. If there was no pelvic adhesion and both fallopian tubes were patent, the diagnosis was standard. Otherwise, it was described as pelvic adhesion or occlusion.

### Statistical Analysis

We used IBM SPSS Statistics for Windows, Version 26.0. (Armonk, NY: IBM Corp) to conduct statistical analysis. Chi-square or Fisher's exact probability method was used to compare differences between groups. *P* < 0.05 was considered statistically significant. Compared with laparoscopy, which was regarded as the “gold standard” procedure for follicular tube examination, the sensitivity, specificity, and positive and negative predictive values of HSG were calculated. Cohen's kappa coefficient analysis was used to evaluate the consistency of the research methodology.

## Results

### The General Characteristic of Patients

A total of 1,276 women with a history of infertility who underwent HSG and laparoscopy were included in this study. The mean age of the patients was 30.67 ± 4.92 (*M* ± *SD*) years (ranging from 18 to 45 years), and the average number of years of infertility was 2.96 ± 2.08 (*M* ± *SD*) years (ranging from 0 to 14 years). Secondary infertility was more frequent (*n* = 863, 67.61%) than primary infertility (*n* = 413, 32.39%), and 20.97% (*n* = 181) of patients had a history of previous pelvic surgery.

### The Comparison of HSG and Laparoscopy in the Diagnosis of Unilateral Fallopian Tube Patency or Occlusion

The HSG and laparoscopic diagnosis results of fallopian tubes are shown in Table 1. When patients underwent HSG, we tended to diagnose right/left side of fallopian tube non-patency. Compared with the right/left tube patency group, the diagnosis of patency/occlusion/pelvic adhesion in the corresponding right/left tube low patency by laparoscopy was significantly different with *p*-values < at 0.007 and <0.001, respectively. Further analysis showed that the diagnosis of occlusion tended to increase by laparoscopy in the right/left tube low patency group by HSG with the rate of 11.2 and 22.8%, respectively. In addition, the proportion of pelvic adhesion was as high as 47.1 and 33.3%, respectively.

**Table 1:** Relationship between the diagnosis of each tube by hysterosalpingography (HSG) and by laparoscopy (*n*, %).

**HSG result**	**Normal**	**Occlusion**	**Pelvic adhesion**	**Total**	**χ^2^**	***p***
Right/not-so-patency	86 (41.7%)	23 (11.2%)	97 (47.1%)	206	NA	NA
Right/patency	109 (50.9%)	8 (3.7%)	97 (45.3%)	214	9.82	0.007^a^
Right/Occlusion	49 (7.9%)	330 (53.3%)	240 (38.8%)	619	19.63	<0.001^a^
Left/not-so-patency	83 (43.9%)	43 (22.8%)	63 (33.3%)	189	NA	NA
Left/patency	109 (59.2%)	15 (8.2%)	60 (32.6%)	184	17.05	<0.001^b^
Left/Occlusion	29 (4.6%)	422 (66.4%)	185 (29.1%)	636	43.84	<0.001^a^

a *Comparison with the not-so-patency tube of the right side*.

b *Comparison with the not-so-patency tube of the left side*.

*NA, non-acquired*.

Compared with the right/left tube occlusion group, the diagnosis of patency/occlusion/pelvic adhesion in the corresponding right/left low patency tube by laparoscopy was significantly different with *p*-values at <0.001 and <0.001, respectively) The cases diagnosed with right/left tube non-patency by HSG tended to be expected and had minor occlusion by laparoscopy compared with the right/left tube occlusion group.

Table 2 shows the performance of HSG in the diagnosis of right tube patency or occlusion compared to laparoscopy as the gold standard. There was a high sensitivity (73.65%), specificity (83.21%), positive predictive value (50.93%), and negative predictive value (92.08%). The Kappa value was as high as 0.47, 95% CI (0.399, 0.541), *p* < 0.001. The corresponding sensitivity, specificity, positive predictive value, and negative predictive value of HSG in diagnosing left tube patency or occlusion were 78.98, 87.72, 56.19, and 95.44%, respectively. The Kappa value was 0.574, 95% CI (0.505, 0.0.643), *p* < 0.001.

**Table 2:** Diagnostic values of unilateral fallopian tube by HSG vs. laparoscopy.

		**HSG (right fallopian tube)**		**Total**	**HSG (left fallopian tube)**		**Total**
		Normal*	**Abnormal^#^**		Normal*	**Abnormal^#^**	
Laparoscopy	Normal*	109	49	148	109	29	138
	Abnormal^#^	105	570	619	85	607	636
Total	214	619	833	194	637	830

* *Normal was defined as patency with the HSG assessment or the laparoscopy assessment*.

# *Abnormal was defined as occlusion with the HSG assessment, or occlusion of single or both tubes, or pelvic adhesion with the laparoscopy assessment*.

### Comparison of HSG and Laparoscopy in the Diagnosis of Both Fallopian Tube Patency and Occlusion

From Table 3, we found that when the bilateral tubes were diagnosed with patency or occlusion by HSG, the probability of bilateral tube patency or occlusion was 87.2 and 58.8%, respectively, which implied that HSG had the same diagnostic value in bilateral fallopian patency as laparoscopy. However, the diagnostic value of bilateral tubal occlusion was relatively poor. The sensitivity, specificity, positive and negative predictive values of HSG in diagnosing bilateral tube patency or occlusion were 97.94, 95.78, 87.2, and 99.38%, respectively (Table 4). The Kappa value was as high as 0.898, 95% CI (0.838, 0.937), *p* < 0.001.

**Table 3:** The relationship between the diagnosis of patency (or occlusion) of both tubes by HSG and by laparoscopy *n* (%).

	**Both tubes occlusion by lap**	**Single tube occlusion by lap**	**Both tubes patency by lap**	**Pelvic adhesion**	**Total**
Patency of both tubes by HSG	2 (1.20%)	0	143 (87.2%)	19 (11.6%)	164 (100%)
Occlusion of both tubes by HSG	282 (58.8%)	129 (26.9%)	3 (0.6%)	66 (13.8%)	480 (100%)

**Table 4:** Diagnostic values of both tubes by HSG vs. laparoscopy.

		**Laparoscopy**		
		**Normal**	**Abnormal**	**Total**
HSG	Normal*	143	21	164
	Abnormal#	3	477	480
Total		146	498	644

* *Normal was defined as patency with the HSG assessment or the laparoscopy assessment*.

# *Abnormal was defined as occlusion with the HSG assessment, or occlusion of single or both tubes, or pelvic adhesion with the laparoscopy assessment*.

### Comparison of HSG and Laparoscopy in the Diagnosis of Hydrosalpinx

Table 5 shows that when the right/left tube was diagnosed as hydrosalpinx, the probability of tube hydrosalpinx was 79.8 and 67.9%, respectively. When the bilateral tube was diagnosed with hydrosalpinx, the chance of real hydrosalpinx was 72.4%, somewhere between the above two probabilities. The remaining was likely to be pelvic adhesion. Regardless of tube hydrosalpinx or pelvic adhesion, both factors contributed to infertility. Moreover, the kappa value of the diagnostic consistency was as high as 0.838, 95% CI (0.754, 0.922), *p* < 0.001.

**Table 5:** Consistency between laparoscopy and HSG in patients diagnosed with hydrosalpinx by HSG *n* (%).

	**Hydrosalpinx by laparoscopic**	**Pelvic adhesion**	**Normal**	**Total**
Right tube hydrosalpinx by HSG	75 (79.8%)	16 (17.0%)	3 (3.2%)	94 (100)
Left tube hydrosalpinx by HSG	72 (67.9%)	24 (22.6%)	10 (9.4%)	106 (100)
Both tubes hydrosalpinx by HSG	97 (72.4%)	36 (26.8%)	1 (0.7%)	134 (100)

*Comparison of the laparoscopic diagnosis consistency between the right and left hydrosalpinx by HSG diagnosis; χ^2^ = 4.73, P = 0.094*.

## Discussion

Exploration of the female genital tract is one of the vital elements of infertility assessment. Laparoscopy provides a comprehensive view of the pelvic reproductive anatomy and a magnified view of pelvic organs and peritoneal surfaces (10, 11). It is generally accepted that diagnostic laparoscopy is the gold standard in diagnosing tubal pathology and other intra-abdominal causes of infertility, such as pelvic adhesion (11–13). Nevertheless, it must be taken in the inpatient department, and the patients need anesthesia. HSG is a frequently utilized diagnostic method in assessing the tubal status and detecting intrauterine anatomical defects in infertility diagnostic patients, which is convenient and safe, and less invasive. To determine the diagnostic value of HSG for infertility factors, we performed this study.

Our study found that the diagnosis of bilateral fallopian tubes as a patent by HSG was very consistent with the diagnosis by laparoscopy. It is reasonable to infer that once the bilateral tube is diagnosed with patency by HSG, the patients have a low incidence of infertility due to tubal factors. However, when the bilateral tubes were diagnosed with occlusion by HSG, there was a 27.5% chance of unilateral or bilateral fallopian tube patency. This may result from insufficiency of contrast agent influx during the angiography operation or spasms of the lower genital tracts. Therefore, the reliability of HSG is always questionable, especially for the diagnosis of tubal occlusion (7, 10, 14). In addition, there were high diagnostic values and consistency of HSG compared with laparoscopy in the diagnosis of bilateral tube patency or occlusion, which was demonstrated by the very high sensitivity, specificity, positive predictive value, negative predictive value, and high Kappa value.

The description of the degree of tubal patency by HSG has critical clinical value and can be divided into no patency, patency, and occlusion (15–17). Compared with the patency group in this study, if the tube was diagnosed with no patency by HSG, the patency or pelvic adhesion in the corresponding tube by laparoscopy was similar. Our results inferred that if the tube was diagnosed as not patent, there was a more than a 40% chance for the tube to be patent. The patients could experience drug treatment or artificial insemination for their next step; in addition, the proportion of pelvic adhesions was more than one-third. Compared with the blockage group, if the tube was diagnosed with no patency by HSG, the diagnosis of patency, occlusion, or pelvic adhesion in the corresponding tube by laparoscopy was significantly different. Thus, we concluded that low patency of the fallopian tube by HSG had a specific guiding significance in infertility analysis. At the same time, we found that the diagnostic values of unilateral fallopian tubes by HSG were high through high sensitivity, specificity, and negative predictive value. However, we still kept in mind the false-positive predictive rate of single tube occlusion. In addition, the diagnostic consistency in occlusion by HSG and by laparoscopy was demonstrated by kappa values of 0.47 [95% CI (0.399, 0.541), *p* < 0.001] and 0.574 [95% CI (0.505, 0.643), *p* < 0.001], respectively, which indicated moderate strength consistency. Considering the low cost and high efficiency of HSG in diagnosing infertility, many scholars recommend HSG as an auxiliary routine outpatient examination in the assessment of infertility (7, 18).

Hydrosalpinx is the morphological change in the fallopian tube resulting from chronic inflammation stimulation (19, 20). Ultrasound and HSG help diagnose hydrosalpinx, but the exploration of hysteroscopy combined with laparoscopy was considered the gold standard for the diagnosis of hydrosalpinx, which could simultaneously inspect the situation of the pelvic cavity (21–23).

Because the peristalsis of the fallopian tube is affected by ovarian hormones (24, 25), it is difficult for ultrasound examination to differentiate hydrosalpinx and severity. With the use of a multidose contrast agent, HSG could effectively diagnose hydrosalpinx (6, 7). In our study, when the tube was diagnosed hydrosalpinx by HSG, there was an ~70% chance accuracy; the diagnostic consistency in hydrosalpinx by HSG and by laparoscopy was very high, and the Kappa value was 0.838 [95% CI (0.754, 0.922), *p* < 0.001]. In recent years, hydrosalpinx has been one of the leading causes of secondary tubal infertility. The diagnosis and treatment of hydrosalpinx significantly impacted the natural conception and pre-treatment of *in vitro* fertilization and embryo transfer (IVF-ET). Considering that hydrosalpinx has a particularly adverse effect on the success rate of IVF-ET, we referred the patient to experience surgical treatment when HSG demonstrated the presence of hydrosalpinx.

Hysterosalpingography has a relatively low expense, but it plays an essential role in predicting the status of the fallopian tube and pelvic situation, which should be conducted as the first diagnostic procedure in assessing infertility. However, the false-positive rate of tube occlusion, correct interpretation of the report, and the course of the procedure could help us make a proper diagnosis. Many strategies could be utilized to overcome the limitations of HSG, including laparoscopy.

## Data Availability Statement

The raw data supporting the conclusions of this article will be made available by the authors, without undue reservation.

## Ethics Statement

The studies involving human participants were reviewed and approved by First Affiliated Hospital of Sun Yat-sen University. The patients/participants provided their written informed consent to participate in this study.

## Author Contributions

JT and YL contributed to the design of the study. JT and MD contributed to a manuscript written and statistical analyses. MX, WP, and ML carried out data acquisition and analysis. YL critically reviewed the study. All authors were involved in and approved the final version of the article before submission.

## Funding

This study was supported by the Natural Science Foundation of Guangdong Province (2021A1515010559) and the Guangdong Provincial Key Laboratory of Reproductive Medicine (2012A061400003).

## Conflict of Interest

The authors declare that the research was conducted in the absence of any commercial or financial relationships that could be construed as a potential conflict of interest.

## Publisher's Note

All claims expressed in this article are solely those of the authors and do not necessarily represent those of their affiliated organizations, or those of the publisher, the editors and the reviewers. Any product that may be evaluated in this article, or claim that may be made by its manufacturer, is not guaranteed or endorsed by the publisher.
